# Supplementary material for: Rummagene: massive mining of gene sets from supporting materials of biomedical research publications
Source: Commun Biol. 2024 Apr 20;7:482. doi: 10.1038/s42003-024-06177-7 (PMC11032387; doi:10.1038/s42003-024-06177-7)
Supplement: Supplementary file 2 — Description of Supplementary Materials [file 42003_2024_6177_MOESM2_ESM.docx]

**Description of Additional Supplementary Files**

**File name:** Supplementary Data 1

**Description:** Rummagene gene sets containing understudied gene sets

**File name:** Supplementary Data 2

**Description:** Rummagene gene sets containing kinases in their table or table-column titles.

**File name:** Supplementary Data 3

**Description:** Rummagene gene sets containing transcription factors in their table or table-column titles.

**File name:** Supplementary Data 4

**Description:** Top 10 predicted genes for terms from GO, GWAS Catalog, MGI Mammalian Phenotypes, and WikiPathways.

**File name:** Supplementary Data 5

**Description:** Cluster 81 gene sets unique to Rummagene.

**File name:** Supplementary Data 6

**Description:** Cluster 116 gene sets unique to Rummagene

**File name:** Supplementary Data 7

**Description:** Cluster 183 gene sets unique to Rummagene.

**File name:** Supplementary Data 8

**Description:** Cluster 225 gene sets unique to Rummagene

**File name:** Supplementary Data 9

**Description:** Commonly detected proteins from mass-spectrometry experiments, highly cited genes, and highly expressed genes.
